# Hogs or Children?

**Published:** 1907-04

**Authors:** 


### Hogs or Children?

In his third article on "Food Adulteration in Texas," Walter B. Whitman says in the November number of *Holland's:*

"Texas has a law, enacted by the last Legislature, which assures purity and full weight of stock feed sold in the State, and adequate provision is made for its inspection. The man who sells adulterated feed for hogs or cattle or sheep subjects himself to heavy penalties. He may sell poisoned milk for the babies, meats filled with borates and sulphites and colored with anilin dyes, preserves and jellies liberally dosed with coal-tar preparations, and any number of other adulterated food for the balance of the family and he goes unpunished. Are the lives of razor-back hogs more precious to the people of Texas than the lives of their children? Do the men of Texas feel more interest in the health of their shorthorn cows than in that of their families?

"If the people of the State desire to have protection against the nefarious practices of adulterators, they can obtain it by insisting on the passage and enforcement of such pure food laws as are now in force in Minnesota, Pennsylvania and other States. While the new National law will tend to correct the existing evils so far as interstate traffic is concerned it will have no effect whatever on the sale of food manufactured within the boundaries of the State."—*Holland's Magazine.*

---

THE ANNUAL MEETING of the Mississippi Valley Medical Association was held at Hot Springs, Ark., November 6, 7 and 8, 1906. The following officers were elected: President, H. Horace Grant, M. D., Louisville, Ky.; First Vice-President, G. A. Hebert, M. D., Hot Springs, Ark.; Second Vice-President, T. C. Witherspoon, M. D., St. Louis, Mo.; Secretary, Henry Enos Tuley, M. D. (reelected), Louisville, Ky.; Treasurer, S. C. Stanton, M. D. (reelected), Chicago, Ill.

Columbus, O., was selected as the next place of meeting, during October, 1907.

It was voted at this meeting to offer a prize of $100 to members of the Association for the best essay recording some original research work in the Mississippi Valley. A committee of three was appointed who will formulate rules of the contest, which will be published later.

